# Supplementary material for: Are carbon emissions trading and green financial instruments synergistic? -Comprehensive quantitative research based on content analysis
Source: PLoS One. 2024 Mar 7;19(3):e0298601. doi: 10.1371/journal.pone.0298601 (PMC10919601; doi:10.1371/journal.pone.0298601)
Supplement: S1 Data — (ZIP) [file pone.0298601.s001.zip › Data/地级市Z_F工作报告.zip!/╔╜╬≈/╠1⁄2╘¡╩╨/└┤╫╘╣1⁄2╓┌║┼í╛┬φ┐╦╩2╛▌═°í┐_2008.docx]

各位代表：

现在，我代表市政府向大会作报告，请予审议，并请各位政协委员和其他列席人员提出意见。

一、2007年政府工作回顾

2007年是“十一五”规划全面展开、重点突破的关键之年。一年来，全市上下在省委、省政府和市委的正确领导下，全面落实科学发展观，实施创新型城市建设主战略，团结一致，开拓进取，圆满完成了各项目标任务。全年完成地区生产总值1255亿元，增长16.4%；财政总收入240.4亿元，增长25.1%；一般预算收入88.4亿元，增长17.4%；规模以上工业增加值497.8亿元，增长28.2%；全社会固定资产投资576.7亿元，增长15.1%；社会消费品零售总额515.9亿元，增长18.2%；地区外贸进出口总额81.1亿美元，增长97.1%；城镇居民人均可支配收入13745元，增长17.1%；农民人均纯收入5561元，增长13.1%。 “六个新突破”和绿色转型启动阶段的目标任务基本完成，城市建设、国企改革和工业经济发展取得明显突破，社会事业和节能减排、生态建设取得重要进展，市政府向全市人民承诺的十二件实事全部兑现！

（一）绿色管理迈出重要步伐，可持续发展能力有所增强

绿色转型标准和政策体系初步建立。把“创新发展模式、推进绿色转型”作为新一届政府的重要执政理念，出台构筑省城绿色产业体系的意见、在经济社会各领域制定和实施绿色标准的意见和2007年绿色考核指标体系。把制定和实施绿色标准作为推进绿色转型的重要抓手，制定和发布了覆盖全市经济社会发展各领域的《太原市绿色转型标准体系》总则和框架部分。目前，太原是全国第一家开始系统制定绿色标准体系的城市。20个部门提出绿色标准初稿，在全国省会城市中率先推出绿色建筑标准。

环境保护与生态建设加快推进。稳步开展绿色企业、绿色社区等“绿色十佳”创建活动。严格实施“绿色高压线”，对58个建设项目予以否决或要求另行选址。大力推进节能减排，实行“区域限批”，治理取缔小机焦，削减焦炭产能252万吨。关停取缔土小污染企业和落后生产设施383个。组织开展百家企业节能行动。完成16户重点污染企业的强制清洁生产审核。燃煤电厂全部实现烟气脱硫。列入国家千户节能企业的13户企业完成节能审计和节能规划编制。新建10.4平方公里“高污染燃料禁燃区”。全力推进超载治理，开展煤焦发运站和煤堆综合整治，市区周边非法储煤场基本清理完毕。大规模推进城市绿化，建成120万平方米的汾河湿地公园，完成玉门河公园、南寨公园和滨河东西路风景林带绿化等道路绿化工程。绿化覆盖率提高1.39个百分点，绿地率提高1.36个百分点，人均公共绿地面积新增0.7平方米。荒山造林4万亩，完成“三北”防护林工程5.4万亩。大力淘汰污染企业和落后生产设施，开展冬季大气环境污染防治百日专项行动，全年减排二氧化硫排放量比上年下降13.8%，空气质量二级以上天数达到269天，市区环境空气综合污染指数下降6.8%。

（二）城市建设拉开框架，“三农”工作持续推进，城乡加快协同发展

城市建设起点高、规模大、效果好。编制完成近期建设规划、城市综合交通规划、13个重点片区规划、7个历史街区景区规划和49个城中村改造修建详细性规划。城建投资完成65亿元，77项重点工程全面铺开。完成龙城大街、迎泽大街及周边微循环、滨河东路快速化改造等一批基础设施重点工程。长风文化商务区等重点片区基础设施建设进展顺利。启动汾河治理美化南延和学府公园建设工程。集中供热扩网759.7万平方米。以煤博会召开为契机，大规模开展城市环境综合整治，完成20余条道路大面积维护和100条道路小修碎补，全面维修道路照明设施，大规模整饰建筑立面，叫停城中村宅基地违法建设1664处。城市建设科技含量不断提高，城市环境面貌进一步改善。

农村经济稳中趋好。加快建立以工促农、以城带乡的投入机制，对种粮农民进行直接补贴。“双千双百惠农工程”任务全部完成，发放支农贷款105亿元。农村经济总收入488.5亿元，增长7.4%。以无公害食品、绿色食品和有机食品为主的生态农业发展迅速，一批农业新品种和新技术得到推广。农业产业化企业实现销售收入52亿元，增长10.6%。完成省级新农村建设试点村规划编制。试点村主要街道硬化、亮化基本完成。农村基础设施建设不断加强，沼气富民工程强势推进，新建户用沼气池1.5万座。新改建农村公路1285公里。治理水土流失面积15万亩。解决了12万农村人口饮水安全问题。扶贫开发成效显著，移民搬迁、整村推进4880人，2.7万人脱贫。

（三）工业经济强势增长，服务业稳步推进，产业结构不断优化升级

工业经济增势强劲。工业总产值完成1628.3亿元，增长49.6%，增速居全国省会城市首位。工业总量在全国省会城市中前移4个位次，居第13位。工业经济运行质量明显提高，规模以上工业经济效益综合指数达到186.9点，比去年提高33点；实现利润93.6亿元，增长70.9%。亏损企业亏损额下降18.3%。循环经济有所发展，资源综合利用水平有较大提高。万元工业增加值能耗下降7.8%。“三百工程”带动作用不断增强，建成投产和在建项目新增产值340亿元，对工业经济增长的贡献率达到60%，拉动全市工业经济增长30个百分点。“三区”集聚效应进一步显现，高新区、经济区、民营区工业总产值分别增长25.1%、63.5%、10.6%。非公有制经济加速发展，完成增加值540亿元，增长18.4 %。

第三产业稳步发展。连锁经营、电子商务、物流配送、特许经营等现代流通业态快速发展。社区商业和农村商业由传统业态加速向现代服务业升级，“万村千乡市场工程”覆盖十县区主要乡镇，建成连锁农家店、农资店736个，农村消费质量和消费规模得到提升。启动放心便民早餐工程。建立完善重点流通企业、重要生产资料市场、肉食市场、农村市场、应急商品数据库等市场监测体系，确保市场供应和食品安全。对外贸易大幅增长，出口商品结构不断优化。不锈钢板材出口增长217%，占出口额比重近三分之一，成为拉动出口的主要力量。加工贸易增长2.3倍。与我市有贸易往来的国家和地区达到161个。旅游业快速发展，实现旅游总收入132.9亿元，增长21.2%。

（四）深层难题逐步破解，改革开放迈出新步伐，自主创新能力不断增强，发展环境继续改善

国企改革取得突破性进展。通过存量转让、增量投入、增资扩股等形式，引进一批战略投资者。南方汽车重组山汽集团，北京中瑞国信和雅世置业重组新凯，中国国电重组南峪煤矿，引进资金120亿元，盘活存量资产20亿元。铜业公司等4户劣势企业实施政策性破产依法退出市场。变压器厂等10户企业列入国家政策性破产计划。晋安东山厂区土地成功转让给富力集团，筹集改制成本近20亿元。2万余名职工通过“退养、补偿、转岗”实现人资分离。债务打包处置取得历史性突破，处置了在金融资产管理公司的58亿元债务。市属商贸企业12亿元的不良债务处置列入商务部减债脱困试点。

财政、人事改革有所推进。实行新的市对县财政管理体制，财力进一步向县级倾斜，提高了基层财政的保障能力。进一步完善部门预算管理，继续推进国库集中支付改革，市级所有部门全部纳入国库集中支付范围，县区试点工作全面推开。按照分类指导、稳步推进的原则，以推行聘用制度和岗位管理制度为主要内容，在建管、教育、卫生、民政等系统开展人事制度改革试点。

科技实力不断增强。全社会研发经费投入23亿元，增长43%。科技成果转化率40%，比上年提高8个百分点。引深技术创新“双十工程”，实施一批以节能减排、绿色转型为重点的自主创新项目，28项技术达到国内领先水平，18个产品填补了国内空白。大力开展产学研战略联盟和产业集群技术联盟试点，中小企业自主创新能力明显提升。完善科技基础条件平台，科技服务能力逐步增强。

招商引资取得成效。立足“绿色招商”，积极组团参加“中博会”、“珠洽会”、“津洽会”、“广博会”、“煤博会”等重大招商引资经贸洽谈活动，履约项目74个，履约资金17.2亿元。新批外商投资企业55户，增长10%；项目总投资27.3亿美元，增长157.9%。实际利用外资增长72.6%。招商引资大项目增多，投资结构优化，引资质量明显提高，发展活力持续增强。

“环境创新攻坚年”活动深入开展。深化行政审批制度改革，将改革重点由减事项转到压环节和办理程序上，将保留的行政许可、行政审批以及服务性事项办理环节压缩30%。构建集中审批新模式，推行并联审批和联合审批。完善基本建设项目“一票制”收费制度，核定建设项目354项。深入推进政务公开，涉及审批事项和对外服务部门的所有审批项目、服务项目、办事程序等全部公开。充分发挥12345便民热线作用，受理来电25.4万个，办结率98.7%。继续开展政风行风评议，对损害政务环境的责任人做出处理。认真受理行政效能投诉举报，按照党纪、政纪条例对相关人员进行处分。巩固扩大创建文明交通活动成果，针对大规模道路拓宽改造，加大交通管理力度，交通秩序得到较好维持。

（五）发展协调性稳步提升，和谐社会建设和特色文化名城建设取得新进展

解决重大民生问题成效明显。认真落实就业再就业扶持政策，城镇新增就业14万人，下岗失业人员再就业 5.6万人，就业困难人员再就业1.1万人。基本解决零就业家庭就业问题。高新区获得“全国模范劳动关系和谐工业园区”称号。促进残疾人在高科技领域就业工作经验在全国推广。城镇登记失业率3.57%。加强重要副食品政府储备体系建设，增加价格调节基金补贴，价格总水平上涨4.1%，低于全国、全省平均水平。社会保险扩面、征缴清欠工作取得新进展，企业养老、城镇职工医疗、失业、工伤、生育保险参保人数均有所提高。各项社会保险基金总收入达到43.2亿元，创历史最好水平。制定实施太原市社保条例，启动城市居民医疗保险。全面推行城市医疗救助制度，救助大病患者1050人。投入3136万元，免除农村义务教育阶段20.7万名中小学生杂费，为3.3万名农村贫困生免费提供教科书，为1.2万人提供生活补助。对5.5万农村低保对象人均补助493元，为五保对象发放供养金570万元。高度关注城市困难群体生活，整体启动涉及近20万人的城市居民棚户区改造工程。经济适用房和安康居住工程竣工83.8万平方米，采煤沉陷区新建住宅50万平方米。对19个地质灾害村进行了集中治理，避让迁村17个，6305人受益。慈善总会开展以“慈善真情暖万家、关注民生大行动”为主题的助医、助学、助残、助老、助孤等救助活动，18万贫困市民受益。整治非法劳动用工，取缔关闭无证砖厂。畅通信访渠道，开通四级联网、覆盖全市的网上信访，信访秩序不断好转。安全生产形势保持平稳，死亡人数下降13.5%。加强社会治安综合治理，有力地维护了省城社会治安稳定，群众安全感明显上升。

特色文化名城建设迈出重要步伐。出台“十一五”时期文化发展规划纲要和加快文化产业发展的意见。初步完成晋祠景区综合改造、蒙山大佛重修和中华傅山园建设。开展纪念傅山诞辰400周年系列活动。晋剧《傅山进京》获“第十届中国戏剧节优秀剧目奖”、“中国戏曲学会奖”，《范进中举》获“第三届中国巴黎戏剧节”“最佳传统剧目奖”。成功举办第九届全国老年合唱节。清徐老陈醋传统酿造工艺和山西中路梆子进入首批国家级非物质文化遗产保护名录。新建一批农村文化活动室。

教育、卫生等各项社会事业发展加快。实施“百校兴学”，新建寄宿制中心小学2所，为64所农村寄宿小学配置暖气，807所农村中小学完成远程教育工程。新型农村合作医疗实现100%覆盖全市所有行政村的目标，农民参合率达到87.2%。扩大农村巡回医疗队和支农专家组对口支援范围，54个乡镇卫生院和906个行政村全部纳入帮扶范围。社区卫生服务机构达到271所，“小病在社区、大病到医院、康复回社区”的医疗服务模式在全省推广。巩固完善济困门诊、济困病房等惠民措施，二级以上医院试点开展7个单病种质量控制和价格公示。人口出生率得到有效控制，自然增长率为4.17‰。第六届城运会取得4金4银2铜的好成绩。我市荣获“全国残疾人体育工作先进单位”称号。外事、侨台、民族、宗教、人防、气象、地震、档案、地方志、国防后备力量建设等工作和老龄、妇女、关心下一代等事业都取得了新成绩。

民主法制建设不断进步。各级政府认真向同级人大及其常委会报告工作，自觉接受人大监督，大力支持政协履行职能，认真接受民主监督。人大代表建议和政协委员提案答复率100%。围绕全市中心工作，认真听取各民主党派、无党派人士和群众团体的意见。积极开展法制宣传和法律援助，全民法制观念逐步增强。圆满完成第三届社区换届工作。深入开展精神文明创建活动，城乡文明程度不断提高，荣获“全省首批文明和谐城市”称号。强化行政监察和审计监督，政府自身建设得到加强，廉政建设取得新成效。

各位代表，过去一年所取得的成绩来之不易。这是省委、省政府和市委正确领导的结果，是市人大、市政协和各位代表、各位委员监督支持的结果，是全市人民团结奋斗的结果。在此，我代表市政府，向辛勤工作在各条战线的工人、农民、知识分子和各级干部，向大力支持政府工作的人大代表、政协委员、各民主党派、工商联、人民团体、无党派人士，向关心帮助太原发展的国家、省驻并单位和驻并部队、武警官兵，向积极参与太原建设的港澳台同胞、海外侨胞和国际友人，表示衷心的感谢！

二、2008年政府工作的总体要求和奋斗目标

各位代表，2008年是全面贯彻落实党的十七大精神的第一年，也是我市全力实施“十一五”规划、深入推进新太原建设最为关键的一年。我市发展面临良好机遇：党的十七大召开后，科学发展观、和谐社会建设深入人心，国家确定山西为全国唯一的煤炭工业可持续发展试点省份，批准我市享受振兴老工业基地政策；省委、省政府进一步明确了山西作为绿色转型大省的战略定位，明确要求太原在全省绿色转型和科学发展方面给全省做出榜样；去年我市经济的快速发展和重大项目建成投运，为今年工作奠定了坚实基础。

也要清醒地看到，我市经济社会发展仍面临着一些深层次问题：一是工业经济面临资源、能源高消耗和投资增长速度有所放缓的双重挑战；二是绿色转型的制约因素比较突出，资源、环境、生态三方面的压力有增无减；三是文化产业和现代服务业比重偏低，发展水平不高；四是投资规模偏低，持续投融资能力不强，投资增长缓慢；五是“城市病”严重，城市规划、建设、管理面临不少矛盾和问题；六是社会事业发展欠账仍然较多，价格上涨压力较大，还有不少影响社会和谐的因素；七是一些部门还存在作风漂浮、执行不力、效率不高等问题。

我们必须认真研究解决这些重大问题，进一步理清思路，努力提高执政能力和工作的科学性、主动性，在工作中坚持以下三个重要原则：一是好字优先，好中求快原则。凡是不符合科学发展的产业和项目，坚决压缩限制；凡是符合科学发展的产业和项目，大力扶持发展，特别要强力推进文化旅游业和现代服务业发展。二是突破瓶颈，因地制宜原则。把国家宏观调控要求与太原实际结合起来，大力优化产业结构和投资结构，增强可持续投融资能力。三是以人为本，改善民生原则。抓紧解决群众密切关注的突出问题，认真落实好 “五大惠民工程”，让改革发展成果惠及全体人民。

2008年政府工作的指导思想是：高举中国特色社会主义伟大旗帜，以邓小平理论和“三个代表”重要思想为指导，深入贯彻落实科学发展观和党的十七大精神，围绕新型工业基地、特色文化名城和现代宜居城市建设，走好“四条路子”，抓好“四大攻坚”，以打造太原经济圈为重点，着力发展现代服务业和高新技术产业，加快产业绿色转型；着力推进节能减排和循环经济，努力转变发展方式；着力统筹城乡发展，积极推进绿色城市化；着力推进改革开放，不断增强自主创新能力；着力改善民生，加快构建和谐社会，建设法治、服务、廉洁、高效、节约环保型政府，推动全市经济社会又好又快发展。

主要预期目标是：地区生产总值增长12%；全社会固定资产投资增长20%以上；工业增加值增长13%以上；社会消费品零售总额增长15%；财政总收入、一般预算收入分别增长15%以上，地区外贸进出口总额增长12%，实际利用外资增长15%。以上为指导性指标。

按照“好字优先、又好又快”的要求，确保完成以下约束性指标：服务业增幅高于地区生产总值增幅；研发投入占全市生产总值的比例达到2.06%；单位生产总值综合能耗下降8.1%，二氧化硫和化学需氧量排放量分别下降7.9%和5.2%；居民消费价格涨幅控制在5%左右；城镇居民人均可支配收入和农民人均纯收入分别增长11%；城镇登记失业率控制在4%以内；农村义务教育保障水平进一步提高，城市义务教育阶段免杂费工作全面实施；人口自然增长率控制在4.2‰以内。

三、2008年政府工作的主要任务

各位代表，新的一年，市政府要坚决贯彻创新型城市建设主战略，着力推进绿色转型，重点做好以下七个方面的工作：

（一）围绕资源节约型和环境友好型社会建设，全力推进节能减排与环境保护

全面加强政府“绿色治理”能力，强制性“抑黑促绿”。加快立法步伐，制定相关政策，构建长效机制。加快推出《太原市促进绿色转型条例》，适时发布实施《反浪费市长令》。制定出台《太原绿色转型标准体系》第三、四部分（实施、评价与改进）、《太原市节水标准》、《太原市绿色机关管理规范》等综合性标准，推动各单位加快制定专业标准，年内确保发布实施10个标准。结合经济、社会和生态实际，以发布实施的重点标准为依据，培育100—200个“绿色十佳”创建目标单位，深入推进创建工作。认真实施省委、省政府制定的地区经济社会发展及和谐山西建设考核评价指标体系。积极探索建立区域绿色转型评价指标体系。加快建立落后产能退出激励机制，加强项目能评和环评审批，实施更加严格的能耗、水耗、环境、安全、资源综合利用和质量、技术等标准。加大“区域限批”和“行业限批”力度，对重点地区和行业实行更加严格的市场准入。整合关闭90座9万吨以下煤矿，将1200余万吨的汽车运煤改为火车运煤。对市内所有工艺落后且超标排污企业和设施进行地毯式排查，限期关停，着手搬迁太化等主要污染源企业和生产设施。

加强重点领域监管，切实推进节能减排工作。大力推进清洁生产，认真实施清洁能源规划，继续对工业企业进行强制性清洁生产审核。组织实施工业企业节能技改工程，万元工业增加值综合能耗下降7.6%。认真执行国家和省关于建筑节能的各项规定，大力实施《太原市绿色建筑标准》，加快研究和强力推广建筑节能的新技术、新工艺、新设备和新材料，加大对新建建筑执行节能强制性标准的监管力度。大力发展节能省地型住宅和建筑。积极推广利用太阳能。改革水资源管理体制，制定水资源综合利用和管理保护规划，出台“十一五”再生水利用规划，推进再生水和中水利用。

贯彻生态优先原则，强力推进生态文明建设和环境保护。严格控制燃煤污染，对2—3年内无法解决集中供热区域内的采暖锅炉强制使用洁净燃料。继续推进城市集中供热，整治城中村燃煤设施，先期治理城中村周边临街营业性质的燃煤锅炉。实施天然气置换工程，置换用户15万户。按照城市污水处理率和垃圾无害化处理率尽快达到100%的要求，加快推进城南污水处理厂建设，对原有污水处理厂进行改建扩建、升级改造，抓好城市垃圾处理场建设。建立危废品处理中心。加快推进城市周围大型防治污染阻隔林带和水污染防护林带建设，突出抓好环城林带建设。继续支持娄烦建设山西生态环保第一县。严格耕地保护，扎实推进太原市第二次土地调查工作。深入开展创建国家园林城市活动，完成漪汾公园、汾河北延湿地公园等重点绿化工程建设任务。抓好城市园林绿化、景区建设和街心公园建设。以环村绿化、村中道路绿化为重点，完成150个村庄绿化任务。全市建成区绿化覆盖率新增1个百分点。

（二）深化经济结构调整，加快构建绿色产业体系

以传统产业新型化和新兴产业规模化为方向，以自主创新为动力，以园区建设为载体，构建具有省会城市特色、辐射带动功能更强的绿色产业体系。

发展循环经济，培育壮大新型工业支柱产业，保持工业经济良好发展势头。制定并实施循环经济总体发展规划，重点抓好新型炉业煤矸石生产耐火材料、太钢粉煤灰蒸压砖等项目。对发展循环经济效果明显的企业给予政策优惠。引深“项目推进年”活动，传统产业新型化实现新突破，突出抓好古交电厂二期、太重智能化起重机、新东方铝业二期、太锅循环流化床锅炉等一批改造项目，使我市传统产业进一步优化升级。新兴产业规模化实现新突破，重点抓好南方重汽、双喜子午线轮胎二期、长城微光器材光纤系列产品等一批高新技术项目，高新技术和新兴产业在全市经济总量中的比重明显提高。以延伸产业链为方向，构筑新的工业支柱。围绕工业经济的绿色转型和可持续发展，出台激励政策，拓宽投融资渠道，有效破解工业投资乏力难题。全市工业总产值突破2000亿元，经济效益综合指数达到195。

以争创循环经济园区和绿色园区为主线，全面改造提升园区经济。理顺园区发展中的体制和机制，做好园区与县区融合规划，促进园区加快发展。高新区要以煤化工产业、创意产业和服务外包产业为特色，加快煤化工技术研发基地、创意产业园和服务外包示范基地建设，积极拓展发展空间，加快与晋源区融合，努力建设全国一流开发区。经济区要做好富士康三期、南方重汽等项目的后续服务，推进生态工业园区建设，加快与小店区融合进程，建设汾东新区。民营区要加快建设工业新区，完善新区体制和基础设施，引导市区工业企业向工业新区搬迁。坚持依托大集团、大企业办园区的方针，积极引进有实力的装备制造、高新技术和新兴产业企业入园。不锈钢产业园区要加速提档升级，全球最大的5万吨无缝钢管项目建成投产，推动太重万吨液压机项目建设。镁生态工业区要尽快完成建设布局规划，推动镁深加工项目进入实质性建设阶段。加快建设汽车工业园，完成第一期工程项目，尽快形成1.5万辆重车生产能力。加快推进西山古交煤焦能源循环经济产业园建设。

改造提升与增量创新并重，加快发展服务业。围绕现代商务中心、区域物流中心、“华夏文明看山西”国际旅游中心、服务全省的金融中心建设，全面制定服务业发展规划，明确牵头机构，整合协调相关部门，统筹推进服务业发展。大力发展以生产性服务为主的研发设计、外贸服务等现代服务业，积极发展物业管理、社区服务、家政服务和社会化养老服务等新兴服务业。以中国太原煤炭交易市场建设为契机，全力推进煤炭现货交易市场的建设和发育。完善城市消费服务功能，启动绿色商业示范工程。积极推动钟楼街、鼓楼街、柳巷、桥头街、开化寺等传统商业街的整体改造和长风商务区、北营区域商业中心等现代商业中心建设。统筹城乡消费需求，抓好“万村千乡市场工程”，完善农村流通网络，改善农村消费条件。按照社会化、专业化、信息化的要求，加紧规划建设北部不锈钢钢材物流园区等重大物流项目，培育物流龙头企业，发展壮大物流业。着力发展旅游业，以打造旅游精品为目标，加快蒙山、天龙山国家森林公园、青龙镇等重点景区景点建设，开发各类“农家乐”旅游项目。完善旅游集散功能，加强与周边城市合作，组织开展“赛在北京、游在山西”主题旅游活动，担当起全省精品旅游线路龙头、全省旅游宣传促销中心、全省游客集散中心这个重任。

加快发展文化事业和文化产业，推动文化大发展大繁荣。大力推进文化产品创作生产，推出一批文化精品，组织好首届“中国太原晋商文化艺术周”大型文化系列主题活动。完善公共文化服务体系，建设太原美术馆、博物馆、北齐徐显秀壁画馆、晋阳古城遗址公园等公共文化设施，加强农村文化活动场所建设，促进城乡文化交流互动。深入推进非物质文化遗产保护和文化信息资源共享等工程，保障群众文化权益。加快文化产业内部各行业间的整合，围绕一批重要文化主题、文化品牌和文化现象，推动文学、戏剧、广播影视、新闻出版、旅游等领域的联动策划和系统开发，实现文化资源的保值增值。降低准入门槛，吸引多元资金投入文化产业。培育打造文广集团等骨干文化企业，形成具有一定规模的优势产业。全面落实文化体制改革试点方案，公益性文化单位要推进“人事、劳动、分配”制度改革，提高公共文化服务能力；经营性文化单位要推动转企改制，提高市场竞争力。加强文化市场管理，营造文明有序的文化市场环境。

坚持把科技进步作为推进产业转型和发展方式转变的第一推动力。推广太钢自主创新经验和做法，引深技术创新“双十工程”，推进产学研战略联盟和产业集群技术联盟。加强国际科技合作，培育一批具有自主知识产权核心技术和名牌产品的创新型企业。全面开展技术发展预测研究，针对经济社会发展中的技术瓶颈，突出节能降耗、污染防治、生态治理等领域的技术攻关，形成一批具有自主知识产权的科技成果。落实支持自主创新的政策措施，加大财政科技投入，鼓励引导企业增加研发投入。组织实施十大科技专项，加快科技资源整合，加速科技成果转化，在做强企业主体、提升产业素质、引领新农村建设、培育科技人才、提高服务能力等五个方面实现新突破。

放手发展非公有制经济。认真落实促进非公有制经济健康发展的各项政策，拓宽融资渠道，建立健全非公有制企业融资机制。非公有制经济增加值增长15%；上缴税金增长12%。

（三）大力推进绿色城市化，加快建设现代宜居城市

按照“山西省省会、承东启西的区域中心城市、山水园林宜居城市、全国重要的新材料和先进制造业基地、具有世界影响力的华夏历史文化名城”的总体定位，高起点规划，高水平建设，高效能管理，努力建设布局合理、设施配套、功能齐全、环境优美的现代宜居城市。采取“政府主导、市场运作”方式，继续加大投资力度，政府投入资本金20亿元，市场化融资100亿元以上，实现城市建设管理的新突破。

坚持“质量第一”，高水平完成重点工程项目。继续做好规划工作，加快完成城市总体规划纲要（2007—2020）修编。加强城市基础设施建设，加快迎泽西大街、大同路、胜利街、汾河公园南延、河西热源厂等16项续建工程进度，开工建设火炬桥、南中环桥、滨河西路快速化改造、晋阳街拓宽改造等一批新的城市道桥工程，集中进行城市小街小巷改造整治。切实抓好城市防洪、污水处理、集中供热等城市配套项目建设。抓好北中环、太行路等4个世行贷款项目。启动长风文化商务区、龙城新区、龙潭公园片区、枣园新区等15个片区的建设。搞好古城保护和开发。全面完成西山棚户区改造任务。健全绿色工程管理体系，做到绿色施工、环保施工、科学施工、文明施工。加大质量监督管理力度，建立质量奖惩激励机制。做好石太高速客运专线、太中银铁路、山西大剧院、图书馆、科技馆、体育中心、会展中心、山西大医院、太原铁路新站等中央、省属重点项目的协调服务工作。

加强规划管理和依法建设，切实加快城中村改造。完善城中村改造的配套政策和文件，制定城中村改造总体规划及城中村改造年度计划，对纳入全市城中村改造名录的148个村实行计划管理，完成重点改造村用地范围的划定和基础数据的收集整理。编制完成9个片区用地控制规划和50个村修建性详细规划。加大对城中村违法建设的整治力度，在城中村集体土地上违法在建、违规销售的项目必须停工、停售。严格实行行政问责制，对落实政令不得力、履行职责不到位、查违成效不明显的单位要通报批评，对参与、支持违法建设和违法用地的干部，要坚决予以查处。

创新城市管理机制，提升城市管理水平。全方位、大力度实施城乡清洁工程，集中整治积存垃圾、卫生死角、占道经营、违章施工和乱吐、乱扔、乱倒行为。对城市出入口、城乡结合部、城市河渠、城中村、居民社区、小街小巷等重点区域和薄弱环节进行重点整治。建立科学严密的市容环境分级巡查体系，实行定岗定人定责管理，分路段、分重点日巡周查。加强公德教育，构建长效机制，倾力打造清洁城市。深化城市管理体制改革，推进管理创新，尽快完成数字化城市管理系统建设，构建作业、管理、执法、监督、评价五个环节有机衔接的高效率“大城管”格局。

加快信息化进程，建设数字化城市。注重用信息化提升、推进新型工业化、绿色城市化和社会现代化。统筹规划、优化整合全市信息资源，构建公共信息平台，实现资源共享。编制全市信息化建设总体规划，抓好政务信息平台整合等信息化应用项目建设，有效提升信息化水平。

各位代表，建设现代宜居城市，是一个宏伟目标。要坚定信心，攻坚克难，使城市建设与管理比翼齐飞，“一年一个样，三年大变样”，城市宜居度和美誉度不断提高，让广大市民为我们的美丽家园而自豪。

（四）统筹城乡协调发展，积极推进新农村建设

加强社会主义新农村建设，发挥试点村和重点推进村的示范带动作用，解决好农业、农村、农民问题，建立以工促农、以城带乡的长效机制，形成城乡一体化发展新格局。

大力发展都市现代农业。以特色农业、绿色农业、品牌农业为目标，优化农产品品质品种结构，重点建设蔬菜、畜牧、林果、小杂粮四大绿色产业基地。规范建设一批规模养殖园区和蔬菜生产基地，巩固提升无公害蔬菜生产基地档次。突出特色、规模、品牌、效益四个环节，实施产业能力提升、休闲服务强化、循环农业构建和产品价值增效四大工程。抓好肉制品加工业产量扩张，带动畜产品产业升级。抓好醋业整合，推动制醋产业扩产增效。大力发展果蔬加工企业，带动果蔬产业增值增效。全市农业产业化销售收入达到55亿元，增长6%；农村经济总收入达到537亿元，增长10%。

加大扶贫开发力度。充分发挥培训基地和农校、农广校、农村中小学等培训资源作用，开展就地培训和就近转移试点，提高农民的专业技术水平和就业能力。积极探索建立生态补偿机制，认真落实“以煤补绿”、“以煤补农”、“以企帮村”的政策和措施。落实“两区”开发项目实施进度，深入开展“关爱老区、帮扶百村”活动，突出抓好整村推进、移民扶贫、劳务扶贫、产业扶贫、生态扶贫和社会扶贫等重点工作，2.7万农村贫困人口脱贫。

切实改善农村基础设施条件和生态环境。以实施“一池四改”为主要内容，加大农村沼气工程建设力度，形成农民家庭内部能流、物流的良性循环，推动农村生产生活方式的根本变革。加快农村路网改造，建设县乡公路350公里，配合省交通厅开工建设太原至古交高速公路。

（五）加快体制机制创新，提高对外开放水平

围绕解决阻碍经济社会发展全局的深层次矛盾和问题，加大攻坚力度，务必在重点领域和关键环节取得新突破。

基本完成国企改革任务。按照“有利于职工就业、有利于产业发展、有利于对外开放、有利于社会稳定”的总要求，一手抓人资分离，一手抓资产运营，集中精力在重点企业、重点问题上攻坚，职工身份置换率、国有产权置换率、改制面三项指标分别达到85%、90%和95%。继续实施“三个一批”战略，做大做强太锅、南汽、狮头等14户企业，完成三晋铝业等4户企业破产重组工作，实施变压器厂等10户企业依法破产工作，对山西电机厂等4户企业实施搬迁改造。支持富力城等开发项目建设。处理好国资系统在金融资产公司的30亿元债务。饮食服务公司所属企业全部实现人资分离。构建国有土地开发、债务处置运营、非主业资产管理服务和离退休人员管理等四大平台，实现国企改革的系统运作。

着力推进投融资体制改革。优化投资结构，破解投资难题，加快投资增速，扩大投资规模。整合现有融资平台，提高资本运作能力。加快市商业银行改革发展步伐。积极推动企业上市，提高直接融资比例。鼓励社会资本投资未禁止的基础设施、公用事业等领域。改革城建投融资管理体制，对城市基础设施等政府性资源实行市场化运作，开发盘活城市存量资产。探索发行城市建设债券，充分利用现有金融机构存款和民间资金。

扎实推进行政、财税管理体制和事业单位改革。按照中央、省行政管理体制改革部署，结合我市实际，积极主动探索和推进政府机构改革，加快建立适应科学发展的新型政府体制机制。推进公共财政体系建设，财政增量继续向新农村建设、社会事业、困难群体生活和公共服务领域倾斜。强化预算管理和监督，完善国库管理体系，扩大政府采购范围。全面实施新的企业所得税法，推进增值税转型改革试点。深化事业单位人事制度改革，以转换用人机制为重点，积极推进岗位管理和人员聘用制。更好地实施人才强市战略，加强人才队伍建设，创新培养、吸引、用好人才的机制，鼓励更多人才在新太原建设中建功立业。

继续扩大对外开放。狠抓“港洽会”、“沪洽会”等签约项目的履约工作，重点抓好一批合同外资1000万美元以上项目的审批和跟踪服务，切实提高资金到位率和项目成功率。组团参加中博会、煤博会、哈洽会、投洽会和高交会等五项赴外招商活动。推进招商“选”资，提高招商质量。发挥比较优势，积极参与中部地区、环渤海地区等区域经济合作。根据国家出口退税政策的变化，抓好出口商品的结构调整，促进出口产品结构向劳动密集与技术、资金密集相结合的方向转化。着力推进“太原经济圈”建设，加强与晋中市及其它周边市县合作，积极探索合作的重点领域、基本途径和有效措施，力争有实质性进展。

（六）全力改善民生，加快发展社会事业，推进和谐太原建设

更加注重社会建设，着力保障和改善民生，促进社会公平正义。以加快教育、医疗、住房、就业、环境管理“五大保障体系”建设为总抓手，大力推进公共服务均等化，努力实现学有所教、劳有所得、病有所医、老有所养、住有所居。

大力发展教育事业。加大“百校兴学”推进力度，每年改造提升百所学校，有效解决优质教育资源短缺问题。经过持续努力，使我们的孩子不仅有学上、上得起学，而且要上好学校，享受优质的义务教育。进一步加强城乡学校交流互动，加快提升农村学校和薄弱学校的办学水平，创建一批省、市示范高中，促进教育均衡发展。推进义务教育标准化建设。新建、改扩建学校基础设施达到或超过国家标准。大力发展职业教育，推进太原大学新区建设，做好新太原师专组建工作。做好农民工子女平等就学和职业学校贫困学生教育资助工作，不能让一个孩子因为贫困而上不起学。

多渠道增加就业岗位。全面落实再就业扶持政策，重点抓好社保补贴、岗位补贴、小额贷款、税费减免等政策的落实。完善面向所有困难群众的就业援助制度。实行创业奖励，支持和鼓励社会各类人员创办中小企业，发挥中小企业促进就业的重要作用。政府继续大力解决“4050”等就业困难人员的就业。就业困难对象再就业8800人。

加快完善社会保障体系。扩大社会保险覆盖面，重点抓好非公有制企业、城镇个体工商户和灵活就业人员参保工作。加大基金征缴和清欠力度，加强社会保险服务和社保基金监管。城镇居民基本医疗保险覆盖面力争达到80%。统筹城镇职工基本医疗保险和城镇居民基本医疗保险，促进相互衔接。企业离退休人员养老金确保按时足额发放。探索建立城市住房保障体系。完善农村最低生活保障制度。加强农村敬老院建设，在全国率先实现农村五保户100%集中供养。逐步开展城市居家养老工作。加大对困难群体的帮扶力度。充分发挥慈善总会作用，加快基层慈善组织建设步伐，广泛开展各类救助活动，让更多困难群众受益。

稳步发展卫生、体育等各项社会事业。大力开展创建国家卫生城市工作。统筹公共卫生医疗服务、医疗保障、药品供应、卫生监管体系的协调发展，加强对传染病的预防控制。新型农村合作医疗保持百分之百覆盖，提高补助标准，农民参合率达90%以上。抓好农村巡回医院的扩建和高效运行，更好地为广大农民服务。深入推进城市社区卫生工作，创建30所星级社区卫生服务机构。加强人口与计划生育工作，稳定低生育水平，提高人口素质。大力发展群众体育运动，抓好奥运会备战工作。办好自行车BMX世锦赛。搞好太原市第二次经济普查和第三次文物普查。以迎接第二届全国创建文明先进城市检查评比为契机，深入开展精神文明创建活动，提高城乡文明程度。人防、气象、地震、档案、地方志等各项工作和老龄、妇女、关心下一代等各项事业，在新的一年都要实现新发展，取得新成绩。

推进民主法制建设。各级政府要定期向人大及其常委会报告工作，自觉接受人大的法律监督和工作监督，主动接受政协的民主监督，高质量高效率办理人大代表建议和政协委员提案。深入开展普法教育，提高全民法律素质。加强基层民主建设，全面启动第八届村委会换届工作。重视做好民族、宗教和侨务工作。加强国防后备力量建设，落实优抚安置政策，增进军政军民团结，继续创建“双拥模范城”。

切实维护社会稳定。深入贯彻《信访条例》，严格落实信访责任制，进一步深入排查化解矛盾纠纷，着力化解重复信访和群体性信访问题。以质量监督、药品监督和食品卫生安全为重点，整顿和规范市场秩序。加强对粮、油、蛋、肉、菜等重要生活消费品的储备管理，保障市场供应。加强对食品、生活必需品、重要生产资料的价格监管，切实稳定市场物价。严格落实安全生产责任制，抓好安全生产和隐患排查治理工作，突出抓好煤矿、非煤矿山、危险化学品、道路交通和城市建设等行业的专项整治。加强对重大危险源的监控，确保安全生产形势持续稳定。加强社会治安综合治理，依法严厉打击各类犯罪行为，引深平安创建活动。建立健全应急管理体系，加强城市应急设施规划和建设，提升城市应急管理水平，提高应对重大自然灾害等突发事件的能力。切实加强人民调解工作，化解矛盾纠纷，努力把不稳定因素解决在萌芽阶段。

（七）积极转变政府职能，提高政府执行力和公信力

创新政府管理理念、管理体制和管理方式，改进作风，狠抓落实，不断优化政务环境。

提高依法行政水平。贯彻《全面推进依法行政实施纲要》，严格按照法定权限和程序行使职权、履行职责，规范行政行为，做到有权必有责、用权受监督、违法受追究、侵权须赔偿。加强行政监督，推行政府绩效管理和行政问责制度，加大对滥用权力、失职渎职和侵权行为的追究力度，严肃整治行政不作为和乱作为现象。严格执行反腐倡廉的各项规定，全面落实党风廉政建设责任制，提高廉洁自律能力。

提高政务服务水平。构建服务型政府基本框架，高效履行经济调节、市场监管职能，着力提供更多更好的公共产品和公共服务。深化行政审批制度改革，清理行政审批的前置条件和标准，减少自由裁量权。把服务与监管职能分开，突出全市各级政务大厅及政务服务机构的服务功能，提高服务水平。进一步完善各级政务大厅“一票制”收费和“一表制”审批制度，开展并联审批和联合审批。建立投资促进服务中心，建成投资政策、行业信息和投资信息数据库，扩大全程代办事项范围，提供优质的行政服务。

提高科学决策水平。建立健全重大决策调查研究、专家咨询、社会听证以及决策反馈纠偏机制和重大决策失误责任追究制度，提升决策的科学化、民主化水平。属于综合性、全局性的决策，要组织跨学科、跨部门和跨行业的专家研究论证。与人民群众利益密切相关的决策，要实行社会公示和社会听证，提高决策的透明度和公众参与度。加强电子政务建设，完善“12345”政府便民服务热线，切实解决实际问题。

提高行政效率和落实能力。强化底线管理，严格实施责任追究制度，确保实现科学发展、构建和谐社会的“安全、稳定、生活、环境和治安”五条基准线。完善各级干部目标责任、业绩考核、末位追究和领导包抓重点工作等管理考核办法，落实重点工作定期督查通报、绩效评议、落实回告等制度，做到任务明确、措施具体、问责追责、奖优罚劣，以科学发展水平论高下，凭科学发展实绩论英雄。

各位代表，解决人民群众最关心、最直接、最现实的利益问题，是人民政府义不容辞的责任。今年市政府承诺办好以下十件实事：一是新建、改扩建和整合100所中小学校。二是城镇新增就业岗位人数12万人，创业就业2.46万人；培训新型农民6.5万人，转移就业2万人。三是县乡村三级医疗卫生机构基础设施70%达标，城市社区卫生服务机构建设完成规划任务的90%以上，搞好国有重点煤矿采煤沉陷区医院的建设与维修。四是加快国有重点煤矿棚户区改造，开工建设住宅11.9万平方米；国有重点煤矿采煤沉陷区治理新建住宅45万平方米；集中治理6个地质灾害严重村，受益1867人；城市居民棚户区改造1万户、40万平方米；开工建设经济适用房51.2万平方米；为廉租住房户提供补贴；解决200户农村残疾人的住房困难。五是市区环境空气质量二级以上天数达到275天。六是新解决10万农村人口饮水安全问题。七是发展连锁农家店180家，扩大放心便民早餐工程。八是发展农村户用沼气池1万座。九是完成通达通畅工程270公里。十是对1620名贫困残疾人实施康复救助，为100个社区配备残疾人康复器材。

各位代表！让我们在党的十七大精神的正确指引下，深入贯彻科学发展观，按照省委、省政府的决策部署，再鼓干劲，乘势而上，圆满完成今年的各项目标任务，为建设服务全省、影响全国、吸引世界的新太原而努力奋斗！
